# Intramolecular Force Mapping at Room Temperature

**DOI:** 10.1021/acsnano.2c09463

**Published:** 2023-01-05

**Authors:** Timothy Brown, Philip James Blowey, Jack Henry, Adam Sweetman

**Affiliations:** The School of Physics and Astronomy, Bragg Centre for Materials Research, The University of Leeds, Leeds LS2 9JT, United Kingdom

**Keywords:** NC-AFM, SPM, submolecular, force spectroscopy, force mapping, single molecule

## Abstract

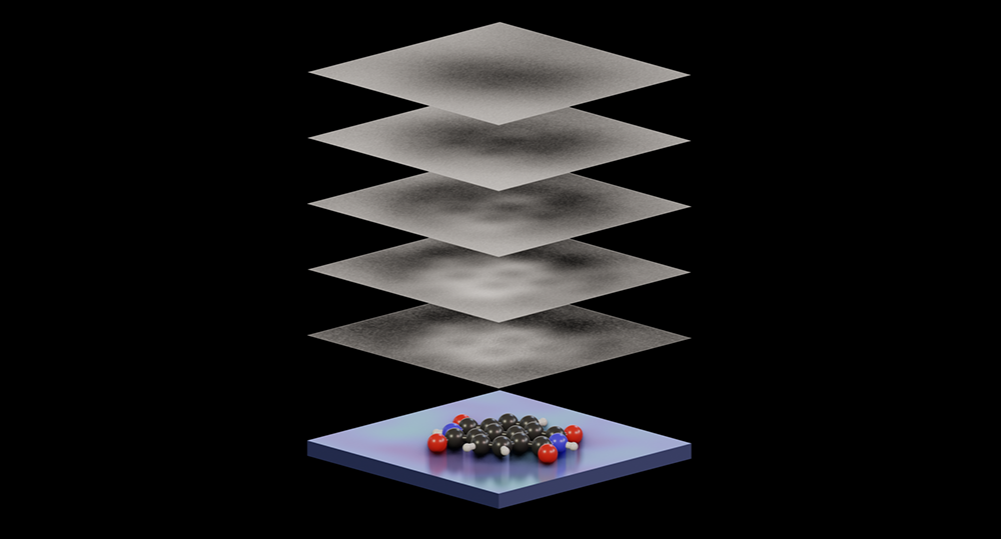

Acquisition of dense, three-dimensional, force fields with intramolecular resolution via noncontact atomic force microscopy (NC-AFM) has yielded enormous progress in our ability to characterize molecular and two-dimensional materials at the atomic scale. To date, intramolecular force mapping has been performed exclusively at cryogenic temperatures, due to the stability afforded by low temperature operation, and as the carbon monoxide functionalization of the metallic scanning probe tip, normally required for submolecular resolution, is only stable at low temperature. In this paper we show that high-resolution, three-dimensional force mapping of a single organic molecule is possible even at room temperature. The physical limitations of room temperature operation are overcome using semiconducting materials to inhibit molecular diffusion and create robust tip apexes, while challenges due to thermal drift are overcome with atom tracking based feedforward correction. Three-dimensional force maps comparable in spatial and force resolution to those acquired at low temperature are demonstrated, permitting a quantitative analysis of the adsorption induced changes in the geometry of the molecule at the picometer level.

## Introduction

The demonstration of submolecular resolution by Gross et al.^1^ showed the unparalleled ability of NC-AFM, using CO functionalized tips, to image the detailed substructure of molecules adsorbed on metal surfaces. Since then, the technique has produced a wealth of impressive results, including the ability to observe bond order in organic molecules,^2^ localization of charge within a molecule,^3^ imaging the stages of a chemical reaction,^4^ molecular manipulation at the single bond level,^5^ and detailed investigation of on-surface synthesized 2D materials such as graphene nanoribbons,^6^ among many others.^7−9^ In particular, many of these results make use of high-resolution spectroscopic measurements, such as high spatial density “force mapping” of single molecules,^10−12^ to elucidate key information about the sample, beyond that obtained via conventional imaging.

Although demonstrating unparalleled resolution and sensitivity, these results have been almost exclusively limited to operation on planar metallic substrates at low temperature (i.e., approx. 5 K via liquid helium cooling). This is due to the twin requirements of both creating and maintaining a functionalized probe by controlled pickup of a single CO molecule (unstable at temperatures above ∼10 K),^13^ and the need for an extremely stable environment with negligible thermal drift (as the tip must approach extremely close to the adsorbed molecule in constant height in order to probe the Pauli repulsion regime).^1^ These practical considerations drastically limit the accessibility of the technique and its use in studying systems under conditions relevant to real-world applications.

It has previously been demonstrated that intramolecular contrast can be obtained on nonmetallic substrates at elevated cryogenic temperatures without deliberate tip functionaliztion,^14−16^ including demonstration of single images showing submolecular resolution at room temperature.^17^ In each of these works, the use of semiconducting materials for both tip and substrate inhibits diffusion of the molecules and can result in tip apexes that are stable for many hours even at room temperature. When repeated observations of a small area (such as a single molecule) are required at elevated temperatures, it is necessary to use feedforward drift correction^18,19^ to compensate for the instabilities caused by small temperature variations. This technique in principle allows the imaging of the same nanometer-sized area for prolonged periods and has previously been used to acquire high density force maps of atomically clean substrates at room temperature.^20,21^

In this paper we show it is possible to reproducibly acquire dense two-dimensional (2D) and three-dimensional (3D) force fields over a small organic molecule adsorbed on the Si(111)-(7 × 7) surface at room temperature. This is accomplished using standard silicon cantilevers and a commercial NC-AFM instrument, without special tip treatment or deliberate tip functionalization. The significant challenges of room temperature operation are overcome via a combination of modified scan protocols and scripted drift correction. The data is shown to have similar spatial and force resolution to data acquired in low temperature environments, and we highlight how acquisition of 3D force data allows a quantitative analysis of the forces responsible for intramolecular contrast, the quality of the probe, and even minor distortions of the molecular structure induced during surface adsorption.

## Results

### Room Temperature Imaging

**Figure 1:**
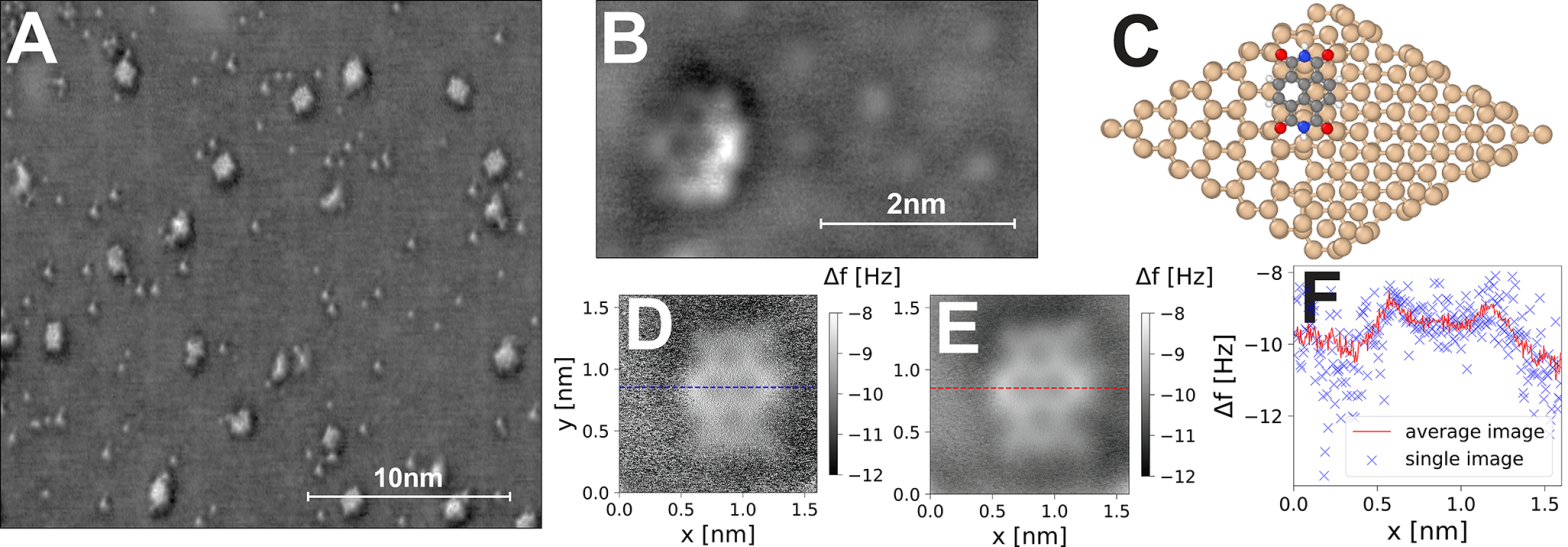
A) AH Δ*f* overview image of NTCDI molecules adsorbed on the Si(111)-(7 × 7) surface at room temperature, image parameters *V*_gap_ = +0.35 V, Δ*f* = −5.2 Hz, Δ*z* offset = −0.23 nm, oscillation amplitude *A*_0_ = 21 nm. B) High-resolution AH image of single NTCDI molecule and substrate atoms. C) Ball and stick model of NTCDI adsorbed on the Si(111)-(7 × 7) until cell, from DFT calculation (color coding: carbon - gray, oxygen - red, nitrogen - blue, silicon - beige, hydrogen - white). D) Constant height NC-AFM Δ*f* image of a single NTCDI molecule at RT (raw data, acquisition time 120 s). E) Averaged constant height NC-AFM Δ*f* image of the same molecule, average of 20 scans (including forward and backward traces), total acquisition time 22 min.) F) Line profile through single and averaged images showing signal-to-noise improvement.

Assessing the feasibility of room temperature intramolecular force mapping required a prototypical molecule–substrate system that was known to be stable at room temperature, and which had already demonstrated amenability to submolecular resolution imaging at cryogenic temperatures. Naphthalene tetracarboxylic diimide (NTCDI) adsorbed onto the Si(111)-(7 × 7) surface has been shown to have a commensurability between the reactive oxygen end groups of the molecule, and the silicon adatom spacing, which allows it to adsorb in an almost planar configuration in a number of sites in the Si(111)-(7 × 7) unit cell, as demonstrated in previous combined STM/NC-AFM studies at cryogenic temperatures.^15,22^ In Figure 1 we show the high reproducibility of intramolecular imaging of this molecule–substrate system at room temperature, which is a vital prerequisite for the significantly more demanding force mapping experiments described later. Figure 1A shows a large scale overview of the surface and the adsorbed NTCDI, acquired in adaptive height (AH). At these imaging parameters the substrate is only weakly resolved, while clear submolecular contrast is obtained on the NTCDI. A number of additional small adsorbates are also resolved, which we assign to adsorbed gas molecules (most likely dissociated OH and H from residual water contamination in the UHV chamber). Figure 1B shows a high resolution AH image taken with a different tip; in this instance both the molecular structure and underlying substrate atoms are clearly resolved and can be directly compared to a ball and stick representation of the system (Figure 1C).

AH imaging is extremely useful for acquiring overview images in the repulsive regime at room temperature as the engagement of the feedback loop on each scan line naturally compensates any residual vertical thermal drift, but reconstruction of a 3D force field acquired by this technique becomes extremely complicated due to the intrinsic misalignment of the absolute *z* position of adjacent spectra.^16^ Acquisition of constant height (CH) data for force mapping is conceptually much simpler, but it requires that the residual vertical drift over the period of a single scan is ≲10 pm. Because fluctuations in ambient temperature result in a continuously shifting thermal drift direction and magnitude, even under optimized conditions this practically limits the maximum acquisition time of a single frame to ∼1–2 min (for details, see the Supporting Information). This can require an increased scan speed and commensurate reduction in signal-to-noise ratio; see Figure 1 D. However, because of the increased resonant frequency of a silicon cantilever compared to a qPlus sensor (typically used at low temperature), we are able to acquire multiple images of the same area and create a low-noise averaged image in a comparable time required to acquire a single high-quality image at low temperature with a qPlus sensor (Figure 1E,F).

We routinely achieve submolecular contrast without deliberately functionalizing our tip apexes. This is in line with earlier low temperature studies, and density functional theory (DFT) simulations, that show that both Si-OH-terminated and Si-terminated probes do not react strongly with adsorbed organic molecules,^17,24^ and find that the stability of the tips is such that they are able to image over a single molecule without tip-changes for ≳30 h. Similarly, we also find that operating at large oscillation amplitudes does not inhibit submolecular contrast, supporting observations in earlier work,^16,17^ and we find that the ultimate limiting factor in our force resolution is the mechanical stability of our commercial room temperature microscope.^25^

A quantitative analysis of the high-resolution images in Figure 1 shows that although at first glance the images fail to show the strong “sharpening” of the bonds and other image distortions associated with imaging with CO terminated tips,^2^ the absolute bond lengths within the molecule are nonetheless exaggerated by ∼10% compared to those calculated via DFT. By comparing the measured bond lengths from Figure 1 to simulated probe particle model (PPM)^26^ images, we estimate that the tip apex in this instance has a lateral stiffness *k*_*xy*_ ∼ 1 N/m (for details, see the Supporting Information), suggesting that our tips have a stiffness between those typically estimated for CO-terminated tips (*k*_*xy*_ ∼ 0.25–0.5 N/m) and the stiffer CuOx tip termination (*k*_*xy*_ ∼ 10 N/m).^27^

### Room Temperature Force Mapping

Although imaging is sufficient for simple characterization of molecular structure for planar molecular systems, in order to identify more complex quantities such as the adsorption angle, distortion in the molecular configuration due to surface–molecule interaction,^11^ or deflection of the tip apex,^12^ it is necessary to acquire grid spectroscopy data in order to allow the direct visualization of the complex force field arising between the tip and adsorbed molecule.

**Figure 2:**
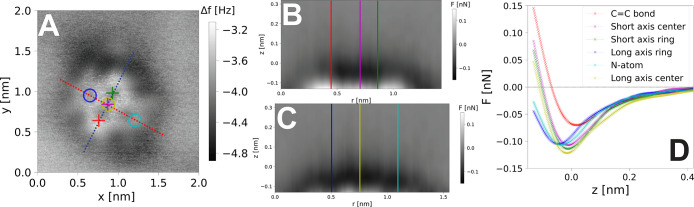
A) CH Δ*f* image of a single NTCDI molecule adsorbed on the Si(111)-(7 × 7) surface, showing the position of the 2D force maps subsequently acquired. Image parameters *V*_gap_ = +0.15 V, Δ*f* = −3.4 Hz, Δ*z* offset = −0.13 nm, oscillation amplitude *A*_0_ = 21 nm. B) 2D force map acquired over the short axis of the molecule (blue line in A)) . C) 2D force map acquired over the long axis of the molecule (red line in A)). D) Representative single point *F*(*z*) curves extracted from both grids showing the force profiles over key sites on the molecule (positions marked on the Δ*f* image and force maps of the same molecule).

To this end, a set of 2D force maps over a single NTCDI molecule were acquired (using the “point spectra” method, see the Supporting Information) as shown in Figure 2. In Figure 2A we show a conventional CH image overlaid with the positions of the 2D grids over the long and short axis of the molecule. These maps are shown in Figure 2B,C respectively, and show the characteristic diffuse attractive halo arising from dispersion interactions surrounding the molecule, as well as the more defined repulsive features due to Pauli repulsion at close approach. The force field over the long axis is roughly symmetric, with a mostly uniform attractive halo and repulsive features at close approach. Figure 2D shows a number of representative *F*(*z*) curves extracted from both grids over key sites on the molecule. The relatively weak minimum (∼100 pN) in the *F*(*z*) curves is indicative of a nonreactive probe interacting primarily via van der Waals and Pauli repulsive interactions. Because the oxygen atoms of the molecule are bound to the surface, there are no strongly reactive sites present, and consequently we only detect a small variation in the force minima over the different areas of the molecule on the order of ∼5–15 pN (for details, see the Supporting Information), demonstrating a comparable sensitivity to similar experiments carried out at cryogenic temperatures. We note here the abnormally small minima on the left-hand side of the molecular short axis (red curve Figure 2D), which arises from a tip asymmetry visible in the force field shown in Figure 2B. The effect of tip asymmetries on quantitative force mapping is discussed in detail in the Supporting Information.

**Figure 3:**
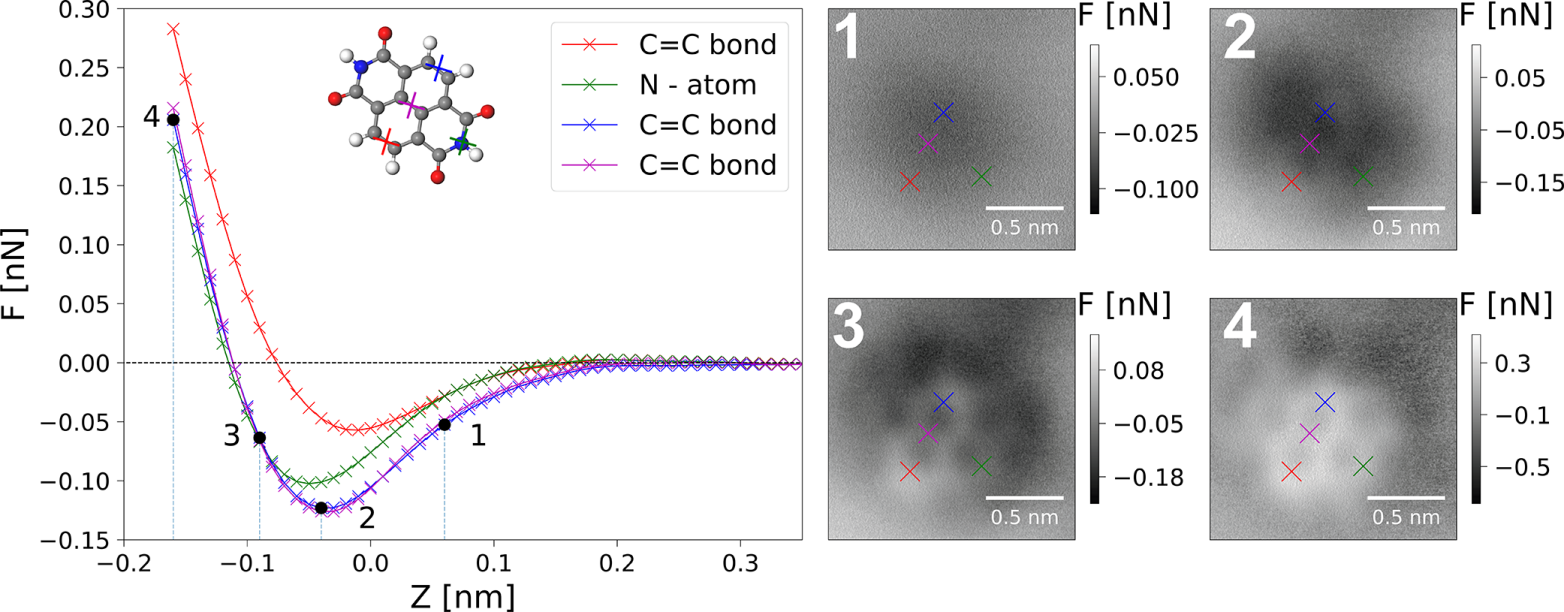
Data extracted from a full 3D force map over a single NTCDI molecule. (Left) *F*(*z*) curves (averages of 10 × 10 pixel area) extracted over key sites on the molecule indicated by crosses on the images and inset molecular model. (Right) *F*(*xy*) images extracted at progressively closer tip-approach at heights indicated on the *F*(*z*) graph. Data acquisition parameters *V*_gap_ = +0.3 V, Δ*f* = −3.1 Hz, oscillation amplitude *A*_0_ = 21 nm.

Two-dimensional force maps, in concert with conventional constant height imaging as demonstrated in Figure 2, provide a relatively quick and convenient way of capturing most of the key information over high symmetry systems and are particularly useful under challenging experimental conditions where acquisition times are limited. However, for more complex systems with a high degree of asymmetry, or where more sophisticated analysis^10,12,28,29^ is required, it is necessary to capture complete 3D force fields, with commensurately longer acquisition times and even stricter requirements for long-term tip and system stability. In Figure 3 we show an example of a 3D molecular force field captured at room temperature, taken on a different NTCDI molecule with a different tip apex, using the “slice” method (for details, see the Supporting Information). In Figure 3, representative *F*(*xy*) images extracted from the 3D data set at decreasing tip–sample heights show a clear evolution in contrast as previously described for similar systems at low-temperature.^14,15^ Representative *F*(*z*) curves extracted from the same data set at key sites over the molecule show a similar variation in minima and force resolution as those extracted from the 2D grids, confirming the correspondence between the two methodologies.

From the force maps we can determine the force sensitivity obtained by mapping the force field over the molecule. Previous room temperature measurements were not precise enough to obtain differences in the tip–sample force with intramolecular resolution, but DFT simulations in the same work predicted a force difference of ∼30 pN between the maximum interaction force over a C atom, and the center of the carbon-ring.^17^ In our measurements we detect a ∼ 10 pN difference between those sites in both the 2D grid data shown in Figure 2 and in the 3D data shown in Figure 3. We note that while the force differences between sites within a single grid were consistent, absolute force values obtained at the *F*(*z*) turning points between subsequent grids on the same molecular site could vary on the order of ∼30 pN (for details, see the Supporting Information), perhaps due to small differences in the tip structure or the location of the background “off” curve used to remove the long-range vdW interactions. Although the interaction is of course dependent on the exact tip structure (which is not defined here) this nonetheless demonstrates that room temperature molecular force mapping has in principle sufficient sensitivity to detect small chemical differences due to single atom substitutions in organic molecules or graphene nanoribbons.^30^

**Figure 4:**
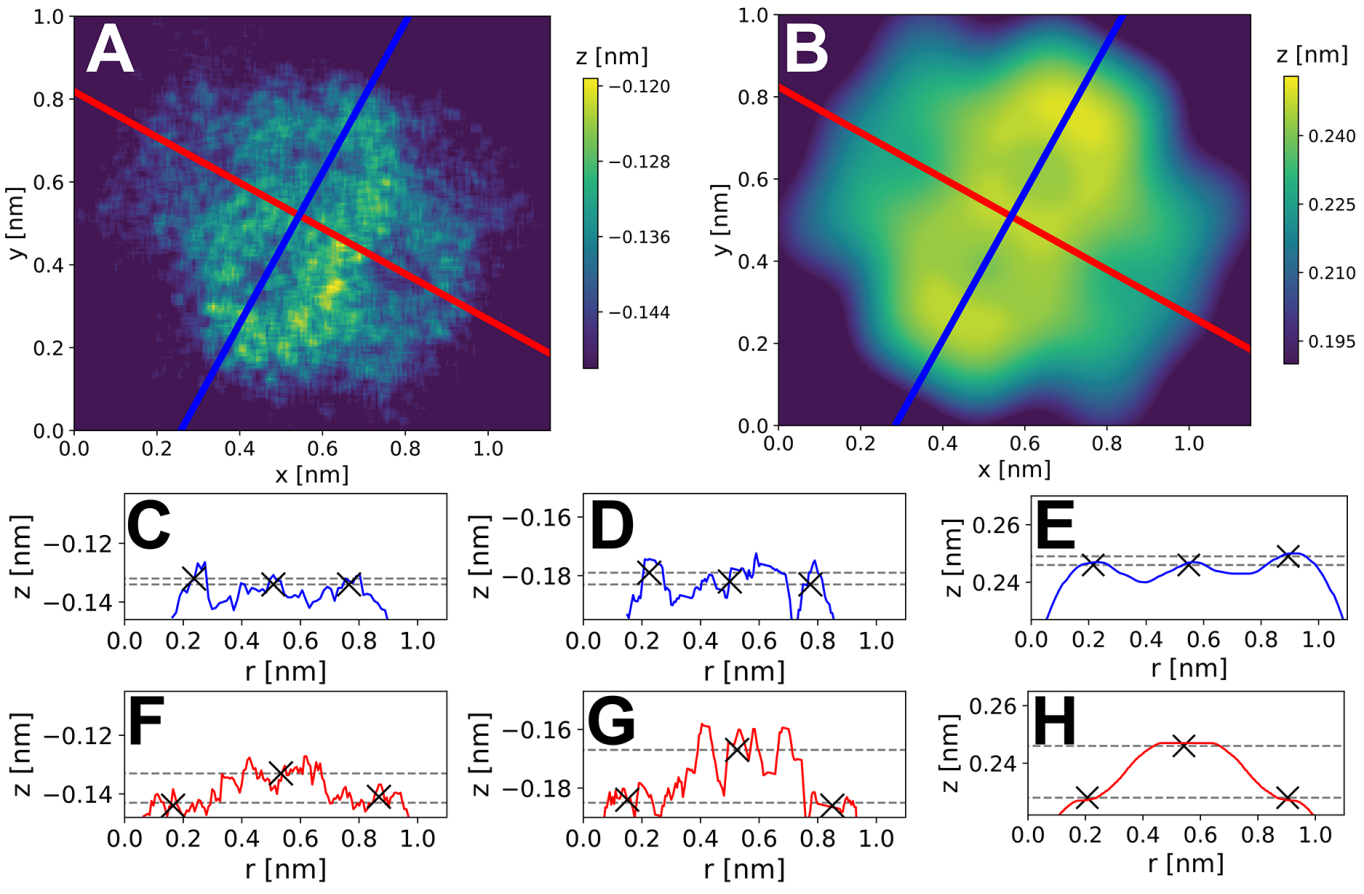
Analysis of structural distortion due to adsorption for a single NTCDI molecule. A) Experimental z* map. B) Simulated z* map. C) Experimental (3D data set), D) experimental (2D data set), and E) simulated, z* profiles through the molecular short axis (blue line in panels A and B), respectively. F–H) As for panels C–E but through the molecular long axis (red line in panels A and B). The extracted heights of the same central and outermost features (marked with an ×) are plotted on each profile as gray dotted lines.

Another key advantage of acquiring higher dimensional force maps as opposed to standard CH imaging is the ability to perform more sophisticated analysis on the spectroscopic data to extract detailed quantitative information about the studied system. In Figure 4, we examine the data set presented in Figure 3, and an additional set of 2D grids acquired on the same molecule with the same tip (for details, see the Supporting Information). We extract the z* position (defined as the vertical *z* height at which the minimum in the Δ*f*(*z*) curve occurs) from each curve to create 2D and 3D z* maps, which have previously been used to extract quantities such as the adsorption angle^11^ and combined topographic/chemical information^28^ of molecular systems. The high sensitivity of these maps reveals additional information about the system, both in terms of the adsorption geometry of the molecule, and the nature of the tip, which must be carefully decomposed.

Due to the symmetry of the molecule we simplify discussion of the data by considering the maps acquired across the long and short axes of the molecule. Turning first to the short axis, it is known that NTCDI adsorbed on Si(111)-(7 × 7) can adopt both planar and tilted adsorption configurations, although from combined experimental and DFT studies it is expected that the planar configuration will be more commonly observed.^22^ In the data sets shown in Figure 4, the tilt of the molecule in the short axis is ∼2–4 pm, as compared to the ∼3 pm tilt predicted from combined DFT and probe particle model (PPM) simulation (for details, see the Supporting Information).^26^ We note that this is flat within our experimental and computational error and intuitively correct as the molecule and adsorption geometry are symmetric with respect to the surface along this axis.

The long axis data in contrast, shows a bowing downward in the z* map, with the end nitrogen atoms being pulled down below the central aromatic rings. In the 3D data set the nitrogen atoms were determined to be 11 ± 7 pm below the central bond of the molecule, whereas for the 2D data set a similar analysis resulted in an estimate of 18 ± 9 pm. This is very similar to the results of the same analysis applied to the simulated PPM data set, which shows a height difference of 18 ± 4 pm (for a full discussion of the analysis and measurement errors, please see the online Supporting Information). Examination of the DFT calculated geometries shows that this molecular distortion arises from the strong bonding of the oxygen atoms at the end of the molecule with the underlying silicon adatoms, due to the almost perfect commensurability between the positions of the carboxylic groups and the adatom positions in this adsorption geometry.^22^ Due to the symmetry of the data and the experimental system, this effect cannot be ascribed to any tip asymmetry, and therefore provides strong evidence that we can quantitatively observe extremely subtle distortions in the molecular framework in our data, despite the extremely challenging experimental conditions. This suggests that structural determination of complex molecules, and even chemical identification of atoms within molecules^28^ is in principle achievable even during room temperature studies.

## Conclusions

We have presented 2D and 3D force mapping of a planar organic molecule at room temperature. The exact conformation of the molecule can be clearly identified using conventional silicon cantilevers with large oscillation amplitudes and without deliberate tip functionalization. Force mapping of the molecules allows direct quantitative analysis of the structure of the molecule, and also assessment of the quality of the tip. Using scripted data acquisition the quality of the intramolecular data achieved is comparable to that previously only obtained at cryogenic temperatures. These results further expand the field of submolecular and 2D material characterization, and suggest the possibility of performing similar measurements under close to real-world conditions.

## Methods/Experimental Section

Data were acquired using a commercial Omicron Nanotechnology VT-STM/NC-AFM, operated using a Nanonis controller. All experiments were performed in ultrahigh vacuum (UHV) at room temperature. The Si(111)-(7 × 7) surface was prepared by standard flash annealing and a low coverage of NTCDI was prepared by depositing the molecules from a homemade Knudsen cell onto the room temperature substrate. Commercial silicon cantilevers (Nanoworld) were treated by argon sputtering to remove the native oxide layer and subsequently prepared on clean Si(111) surfaces via gentle indentation to the surface until good NC-AFM resolution was achieved. Cantilever deflection was measured using a standard four quadrant photodetector, rather than an optical interferometer as described in previous cantilever studies,^17^ but this did not impede our ability to obtain submolecular contrast. NC-AFM imaging was performed with a small voltage applied to the tip (typically 300–400 mV) to remove the electrostatic force due to the contact potential difference (CPD). Due to the semiconducting nature of the tips, no tunnel current was observed throughout the experiments, nor did we use any scanning tunnelling microscopy (STM) based techniques to prepare the tip apex. Atom tracking was used to apply feedforward correction for imaging and for 3D data acquisition. All *z* heights are given relative to the tip height over the molecule at the frequency shift (Δ*f*) feedback value used for feedback during atom tracking. Site-specific forces were extracted from Δ*f*(*z*) spectroscopy curves using the Sader–Jarvis algorithm^31^ after subtracting the non-site specific forces by measurement of the tip–sample interaction over a corner hole site.^32,33^ All NC-AFM imaging was performed in constant height (CH) mode, except for large overview images, which were performed using a multipass scan mode introduced by Moreno et al.^16^ (referred to as adaptive height (AH) mode in this manuscript), which uses an intermittent disengagement of the feedback loop to obtain pseudoconstant height images in the repulsive regime on samples with topographically different regions.

## Supporting Information Available

Additional supporting experimental data sets, discussion of tip asymmetry effects, simulation details, data processing steps, and discussion of the influence of thermal drift (PDF)
Video versions of Figures 3, S7, and S9 (ZIP)
