# Supplementary material for: Intramolecular Force Mapping at Room Temperature
Source: ACS Nano. 2023 Jan 5;17(2):1298–304. doi: 10.1021/acsnano.2c09463 (PMC9878970; doi:10.1021/acsnano.2c09463)
Supplement: Supplementary file 1 — nn2c09463_si_001.pdf [file nn2c09463_si_001.pdf]

Intramolecular force mapping at room temperature: Supporting information

Timothy Brown*, Philip James Blowey, Jack Henry, and Adam Sweetman*

The School of Physics and Astronomy, Bragg center for Materials Research, The University of Leeds, Leeds, LS2 9JT, U.K.

Methodology

Data were acquired using a commercial Omicron Nanotechnology VT – STM/NC-AFM, operated using a RC5 Nanonis controller and PLL (OC4). All experiments were performed in Ultra High Vacuum (UHV) at room temperature. The Si(111)-(7×7) surface was prepared by standard flash annealing at 1200 °C and a low coverage of NTCDI was prepared by depositing the molecules from a home-made Knudsen cell. Commercial silicon cantilevers were treated by argon sputtering for 5 minutes (with 1×10^{-5} $mbar$ of Ar^{+} and beam energy 2 kV) to remove the native oxide layer, other contaminant elements, and also sharpen the tip, The tips were subsequently prepared on clean Si(111) surfaces via gentle indentation to the surface until good NC-AFM resolution was achieved in a similar manner to Iwata et al. (2015) [1].

Nanoworld cantilevers manufactured with spring constant (k) $48N/m$, resonant frequency (f_0) $170.021kHz$, were used in the NC-AFM experiments. Large oscillation amplitudes (A_0) were used ~ 20 nm. Cantilever deflection was measured using a standard four quadrant photodetector, rather than an optical interferometer as described in previous cantilever studies [1], but this did not impede our ability to obtain intramolecular contrast. NC-AFM imaging was performed with a small voltage applied to the tip (typically 0.3 - $0.4V$) to remove the electrostatic force due to the contact potential difference (CPD). Due to the semiconducting nature of the tips, no tunnel current was observed throughout the experiments, nor were scanning tunnelling microscopy (STM) based techniques used to prepare the tip apex.

In order to maximise thermal stability, the temperature in the microscope head was maintained slightly above room temperature (21 – 23 °C) using a Lakeshore 331 temperature control unit connected to a heater on the sample stage. The parameters for the feedback mechanism were optimised to reduce temperature fluctuations (which significantly influenced the thermal drift) until the temperature stability reached $\pm 2mK$. The chamber was also shielded using UHV bake-out cladding, to minimise the influence of variations in the laboratory temperature.

Lateral feedback electronics and lock-in amplifier were provided in the Nanonis controller atom tracking module. With the tip locked onto a surface feature [2], an average position of the path it traced over a few seconds was calculated. Following iterative measurements, one can determine its displacement over time, and hence apply a feedforward correction vector

to the piezo scan-tube to compensate. To account for the non-linear nature of thermal drift / piezo electric creep in scanning probe microscopy (SPM), the feedforward vectors need to be updated with some level of regularity (see section on positioning error below).

Home-built LabVIEW scripts were used, which pause and resume the atom tracking in between data acquisition. Multi-dimensional Δf data sets were acquired via two principal methods, grid spectroscopy [3, 4] and stacking constant height scans [5, 6]. Each method should, in principle, produce the same data. However in practice they each have different strengths and weaknesses.

When gathering 3D data sets, with comparable data densities, both methods require lengthy overall acquisition times (8+ hours). However, when comparing the duration between tracking events of each method, grid spectroscopy has an advantage. For the grid spectroscopy, a single element (one curve), may be carried out (with acceptable signal-noise ratio (S/N)) of the order of a few seconds, whereas a complete CH image in NC-AFM typically takes longer, of the order of 1-2 minutes. The larger acquisition time of scanning compared to point spectra is a constraint on the frequency of drift corrections made. The larger acquisition time also increases the uncertainty in z-position for scanning, compared to point-spectra. However, the slice method boasts a fully retrievable data set even if a tip change occurs during close approach to the molecule. A tip change during grid spectroscopy is likely to result in an incomplete data set as the tip approaches to the closest position for each pixel in (x, y) during the measurement.

The parameters used in all grids are as follows: For Fig.2 in the main text (2D grid): 300 point spectra, each of 512 points, spanning 630 pm in z, across 1.8 nm in the (x, y) plane. The script was first conducted over the short, then long axis of the NTCDI molecule. For Fig.S5 (2D grid) 400 point spectra, each of 512 points, spanning 700 pm in z, across 2.0 nm in the (x, y) plane. For both grids, data acquisition was paused after every 5 spectra to resume atom tracking. For Fig.3 from the main text: a series of constant height images of scan size 1.6 nm^2 was taken, spanning 660 pm above the molecule. The increment in z was 10 pm. Each height was scanned three times, in order to take an average, to increase the S/N, in the same manner as Fig.1E in the main text. Data were processed with custom Python code and Gwyddion [7].

Adaptive Height Mode

In preparation for the scripted experiments on single molecules, larger (> 10 nm) overview scans were conducted in adaptive height mode (introduced by Moreno et al. (2015) [8]) in order to locate the NTCDI molecules, as well as evaluate the tip state for potential further tip reconfiguration [1]) and the cleanliness of the surrounding substrate.

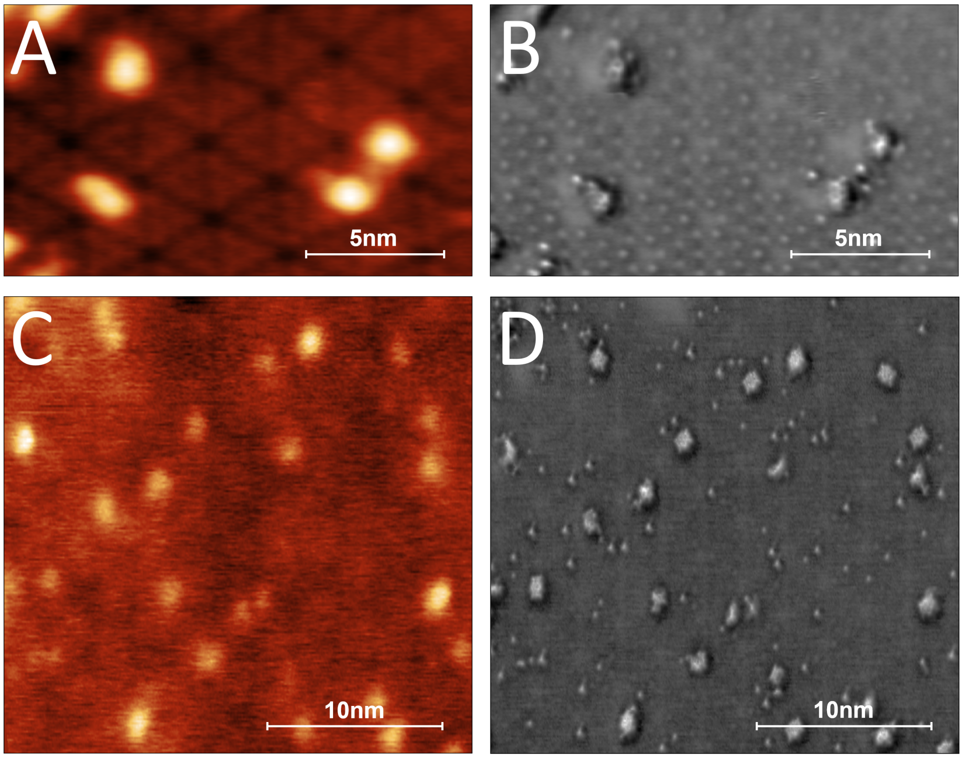

Supporting Figure 1: Two examples of scanning in adaptive height mode NC-AFM, of NTCDI molecules deposited on a Si(111)-(7×7) surface. A) & C) are topography images with Δf set points $-0.9\ Hz$ and $-3.6\ Hz$ and gap voltages, V_{gap} $+0.45\ V$, $+1\ V$, respectively. B) & D) are the respective corresponding Δf channels recorded without feedback in z. The offsets in z, when measuring in constant height mode, for B) & D) are $-0.192\ nm$ and $-0.3\ nm$ respectively.

In adaptive height (AH) mode, each line in the slow-scan direction is scanned twice, first with Δf feedback applied, to maintain the tip-sample distance. This is followed by a scan without feedback. However this second pass traces the line profile of the first pass. This ensures the tip remains at a suitably safe distance from any large features/contaminants. In order to get closer to the surface, an additional offset to the tip height can be added to the pass with feedback disengaged. Adaptive height mode allows close approach of the surface features in order to observe intramolecular contrast whilst mitigating the risk of a tip crash due to thermal drift, as feedback is resumed after each line in the fast-scan direction.

Tip Characterisation

Characterisations of the experimental tips on the silicon adatoms were not routinely performed [6]. However we did obtain AH images (see Fig.S2), where atomic resolution over the substrate and submolecular resolution over the NTCDI was achieved simultaneously.

In this image, the repulsive contrast over silicon adatoms indicates the tip was passivated, potentially terminating with a OH^- group, which has been observed to improve contrast [1]. However we note that both reactive and unreactive silicon tips have shown the ability to image with submolecular resolution [1, 6, 9].

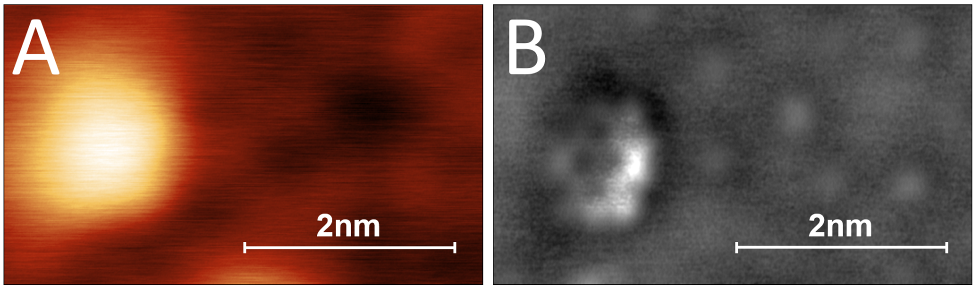

Supporting Figure 2: Image from Fig.1B Adaptive height mode image, of a single molecule, showing Z and constant height Δf channels. The positions of the substrate adatoms are resolved alongside intramolecular contrast over the NTCDI. Imaging parameters $V_{gap} = +1\ V$, $\Delta f = -0.8\ Hz$, Δz offset $= -150\ pm$.

Estimating Tip Apex Stiffness

The lateral stiffness, k_{xy} of the tip apex determines the degree to which it will deflect under strong tip-sample interaction. This deflection results in the exaggeration of image features, i.e. the individual bonds of the molecule [10]. A CO molecule adsorbed on the tip apex has been shown to have a low lateral stiffness [10–12], and thus deflect laterally to exaggerate bond lengths.

Using the same method as Mönig et al. (2018) [10] (see Fig.S3), we calculated an estimate for k_{xy} by comparing the bond lengths from experiment, and data from PPM simulation of the calculated DFT structure. The average exaggeration in bond length as a % was calculated across a range of simulated stiffness values. A simulation using CO-level stiffness ($k_{sim} = 0.5\ N/m$) resulted in a bond length exaggeration of 15%, whereas for a CuOx tip ($k_{sim} = 10\ N/m$), this exaggeration was 2%. The experimentally determined bond lengths are on average $\sim$ 10% larger than DFT prediction. We found the best agreement between experiment and simulation using $k_{sim} = 1\ N/m$, and tip charge $Q = 0$, which also yielded an exaggeration of 10%. Therefore we propose this is the approximate stiffness of the experimental tip, whose value lies between that of CO tips and CuOx tips.

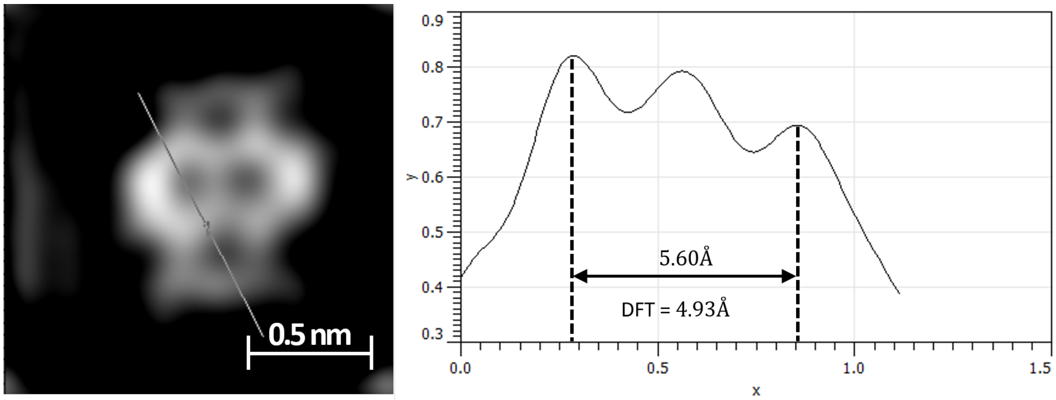

Bond measurement		
Distance	Length(Å)	% exaggeration
Two rings	5.60	11
Two rings	5.35	8
Two rings	5.57	12
Two rings	5.42	11
Short axis	5.69	11
Long axis	7.70	10

Supporting Figure 3: Measurements of principal axes and distances across C=C bonds were conducted over average of 20 images from Fig.1E (main text). Line profiles were drawn across by eye in Gwyddion (example shown left). The distance between maxima were calculated from the drawn profiles (shown right). Each profile was compared to its counterpart in the DFT structure (see below). The average increase in bond length was calculated to be 10.5%.

Data Processing

2D Grids

In the case of the 2D grid spectroscopy files, we sometimes observed a slow drift in the resonance frequency, f_0 of the cantilever. We believe this to be a result of thermal fluctuation of the cantilever, or the reference oscillation of the PLL [13]. The full 2D Δf grid shown in Fig.S4 reveals that across 400 spectra files (representing 2 nm), the tail end of the Δf signal in the long range interaction regime systematically shifts ~ -0.1 Hz from the first curve to the last over the course of the experiment (left to right).

In order to prevent the distortion of the subsequent inverted $F(z)$ data, the $\Delta f(z)$ curves were shifted in Δf in order to align the long range part of the curves with that of the first curve in the sequence. This was done using a linear regression to minimise the residual Δf shift between subsequent curves in the sequence. As a consequence, the distortion was removed from the converted force data.

Additionally, the 2D Δf grids were smoothed in xz using a 10 pixel-wide averaging window.

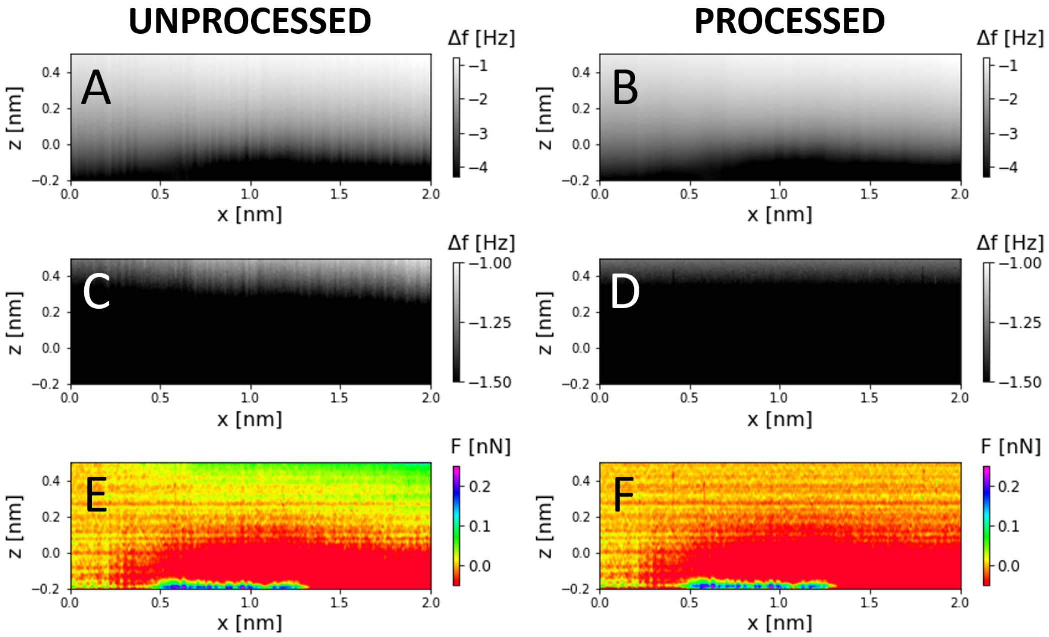

Supporting Figure 4: Processing of 2D grid spectroscopy data: A) raw Δf. B) Δf following processing via a 10 point rolling average in x, and alignment of the long range data in Δf. C) & D) as above but with colour bar of figure adjusted to highlight the behaviour of the Δf at large tip-sample separations. E) & F) are the outputs of the force inversion from the unprocessed and processed data sets respectively.

Force Inversion

The background signal (long range interactions) was removed by subtracting $\Delta f(z)$ curves taken over the bare substrate, (also known as an off-curve [14]) from each $\Delta f(z)$ curve in the grid. For the grid spectroscopy data sets, the first curve was taken to be the background and was subtracted from all other curves. The short range Δf signal created was then converted into the vertical tip-sample force using the Sader-Jarvis algorithm [15]. After conversion, the $F(z)$ data were smoothed in z using the splrep function from the scipy.interpolate python library (smoothing parameter $\lambda = 0.99$). Single point $F(z)$ curves were extracted from the data having been averaged in x with a 10 pixel wide averaging window.

3D Grids

For the slice method data set, constant height images recorded at different z heights were stacked to make a 3D grid. The repeat scans at each height were averaged to improve the S/N ratio and alignment of the molecule in the (x, y) plane. No $post - hoc$ alignment of the constant height images was carried out for Fig.1 and Fig.3 in the main text, as image alignment was already sufficient due to feedforward compensation.

Force Inversion

The $\Delta f(z)$ signal in the upper right hand corner was used as the background signal, and subtracted from the rest of the grid. The force conversion was then carried out using the Sader-Jarvis algorithm [15] on the resulting short range $\Delta f(z)$ signal across all points in the (x, y) plane. The force data was then smoothed with a spline fit in z ($\lambda = 0.99$). The force grid was smoothed in (x, y) using a 10×10 pixel sized averaging window. The single point $F(z)$ curves were extracted from this averaged data, with a further 10×10 averaging.

Extraction of $z^*(x, y)$

We adopted the method used by Mohn et al. (2011) [16, 17] to map the minimum Δf or force value for each point in (x, y) across the molecule:

$$z^*_{\Delta f}(x, y) = \arg\ \min \Delta f_z(x, y, z) \tag{1}$$

$$z^*_F(x, y) = \arg\ \min F_z(x, y, z) \tag{2}$$

Before $z^*_{\Delta f}(x, y)$ extraction, the Δf images were first smoothed with a rolling average in the (x, y) plane with a window size of 20×20 pixels. The Δf data was then smoothed in the z direction using a spline fit at each point in (x, y) ($\lambda = 0.99$). The minimum value in $\Delta f(z)$ at each point in $(x, y) : z^*(x, y)$ was then extracted. The 2D $z^*(r)$ profiles, however, were extracted without any smoothing of the raw data. The profile itself was then smoothed with an averaging window of 3 pixels.

Supporting Data Sets

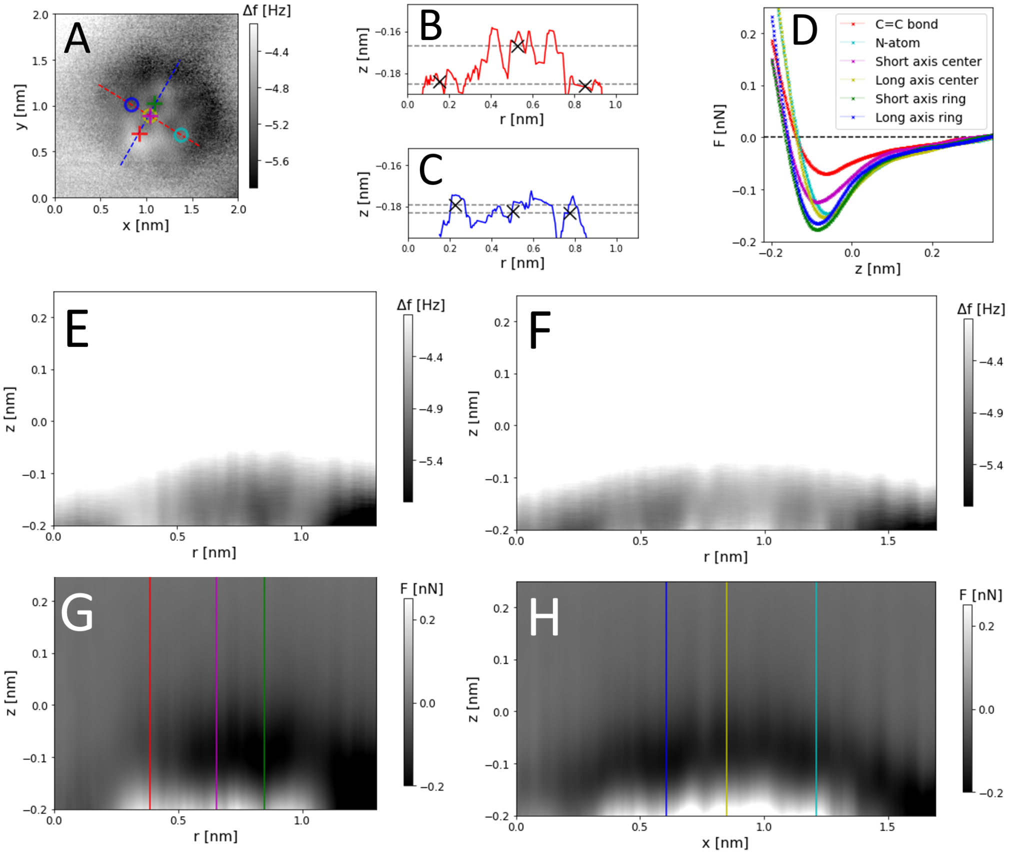

Supporting Figure 5: Additional 2D grid spectroscopy data set. A) Constant height Δf image of molecule preceding experiment. Image parameters $V_{gap} = 0.3\ V$, $\Delta f = -3.1 Hz$, oscillation amplitude $A_0 = 21\ nm$. B) & C) profiles of the $z^*(x, y)$ across the short and long axes respectively, taken via 2D grid spectroscopy. The average heights of features are marked with $\times$. D) Single point $F(z)$ curves taken from the 2D force map over sites indicated in A), G) and H). E) & F) raw Δf data across the short and long axes of the molecule respectively. The colour scale has been shifted to increase contrast across the sites of the molecule. G) & H) Force data across the short and long axes of the molecule respectively.

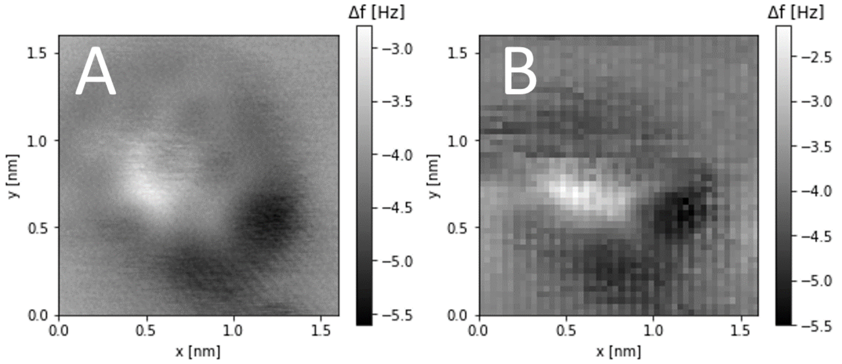

Supporting Figure 6: 3D maps taken via slice and grid methods over 1.6 nm^2 area containing a single NTCDI molecule. Following the constant height slice method A) (256 $\times$ 256 pixel images across 650 pm in z, in increments of 10 pm), a 3D grid spectroscopy experiment was attempted. B) (48 $\times$ 48 grid density with each $\Delta f(z)$ curve sweeping 1024 points across 650 pm in z). A tip change occurs part of the way through the experiment after 24 hours measurement, possibly due to repeatedly taking the tip to the point of closest approach.

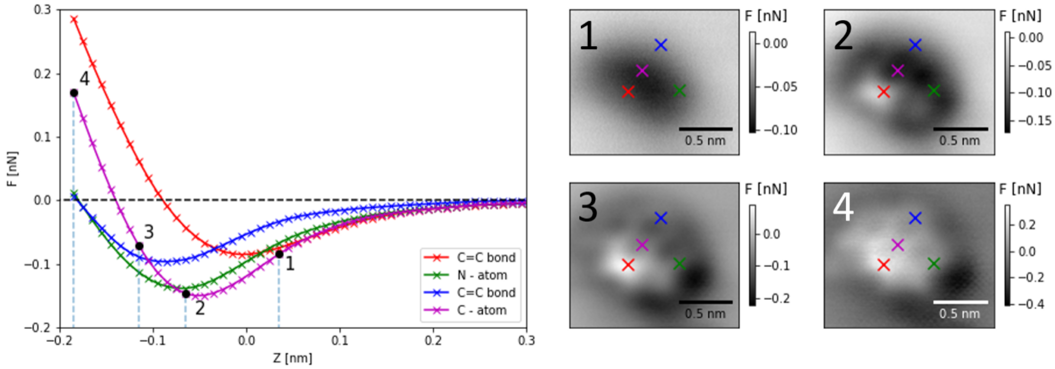

Supporting Figure 7: 3D force map constructed using the data acquired via the slice method in Fig.S6. Left: $\Delta F(z)$ curves extracted over key sites on the molecule indicated on the images to the right. 1), 2), 3), & 4) (x, y) images extracted at progressively closer tip-approach at heights indicated on the $\Delta F(z)$ graph. Data acquisition parameters $V_{gap} = +0.45\ V$, $\Delta f = -4.8\ Hz$, Oscillation amplitude $A_0 = 21\ nm$.

Further data sets / force maps are provided here to demonstrate the reliability of the experimental protocol.

Fig.S5 shows the 2D grids performed over the same molecule, and the same tip, as used in the 3D data set in the Fig.3 (main text). The raw Δf grid data is also plotted, with the colour bar adjusted to reveal the variation in signal across different sites of the molecule. Fig.S5 B) & C) are the same profiles of $z^*(x, y)$ seen in Fig.4 in the main text.

Fig.S6 depicts Δf (x, y) plane images of a 3D data set constructed by the slice method

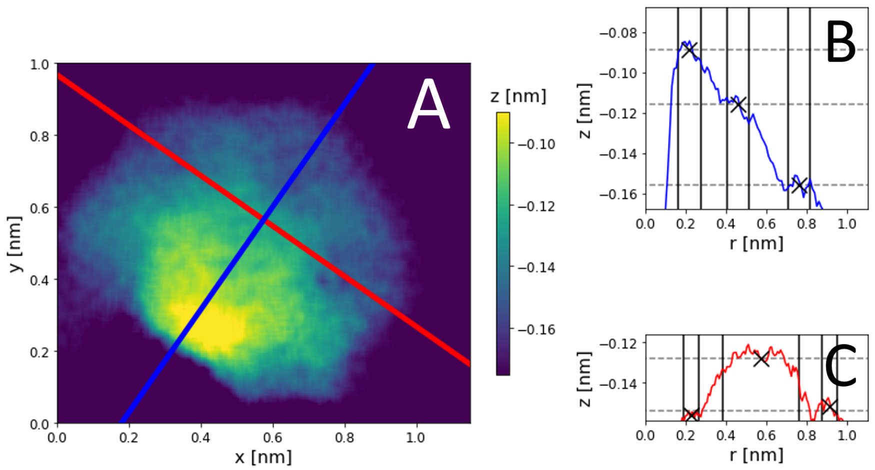

Supporting Figure 8: Extracting the force map data from Fig.S7 taken using the slice method, the A) $z^*(x,y)$ image, and line profiles through the short and long axes of the molecule B) & C). The heights across the features are calculated and indicated by the X markers (using data ranges indicated by the vertical lines in B) & C)).

A) and then grid spectroscopy B). The acquisition time for the slice method was $\sim$ 8 hours, and $\sim$ 48 hours for the grid spectroscopy. Approximately halfway through the grid spectroscopy method there was a tip change, thus giving a discontinuity in the grid and subsequent resolution of the molecule.

Using the slice-based 3D Δf map from Fig.S6A, $F(z)$ curves and (x,y) showing the evolution of the contrast are plotted in Fig.S7 (in the same manner as Fig.3 from the main text). Moreover, the $z^*(x,y)$ can be extracted from the 3D Δf matrix, and the heights of different sites of the molecule were determined and plotted in Fig.S8 (in the same manner as Fig.4 from the main text). Along the long axis, the nitrogen atoms are positioned on average 22 ± 8 pm below the central carbon double bond, and is in good agreement with measurements performed on other tips, thus suggesting only measurements made along the direction of the asymmetry are affected.

Simulated AFM Data

Using the probe particle model (PPM) [18], simulated AFM images were generated to construct a similar 3D force map as in the experiment. Given the unknown exact geometry of the tip, a range of probe stiffness, k, and charges, Q, were tested. For the data presented we used the set of parameters which most closely recreated the experimental bond length (see section on tip stiffness), $k = 1$ N/m and $Q = 0$. The apparent bond length in the resultant PPM images was on average 10% larger than in the DFT geometry, similar to the experimental exaggeration of 10.5%.

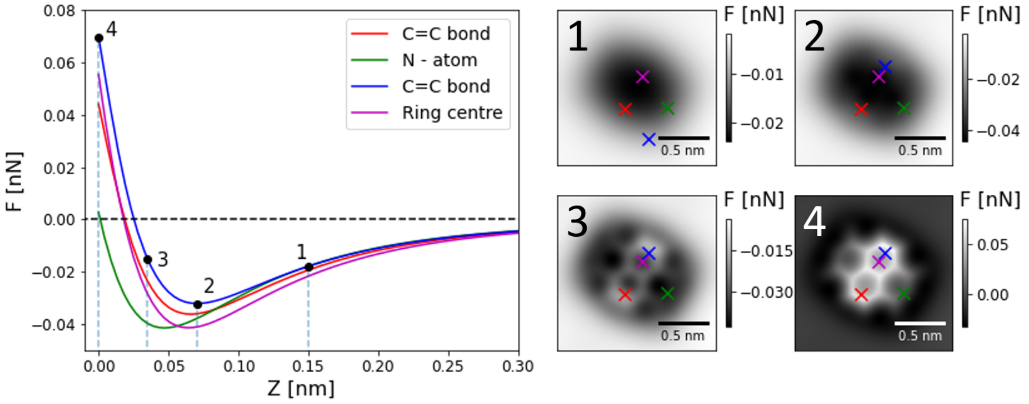

Supporting Figure 9: Map of the force from PPM simulation, spanning 0.3 nm above the NTCDI on Si(111)-(7×7), with a vertical spacing of 1 pm. Left: Single point force spectra from the 3D data set, with positions in (x, y) indicated by the markers in images 1), 2), 3), & 4) which show cross-sections of the 3D force map in the (x, y) plane at various heights, represented by the numbers 1) - 4) in the graph. $k = 1$ (N/m), $Q = 0$.

DFT Calculations

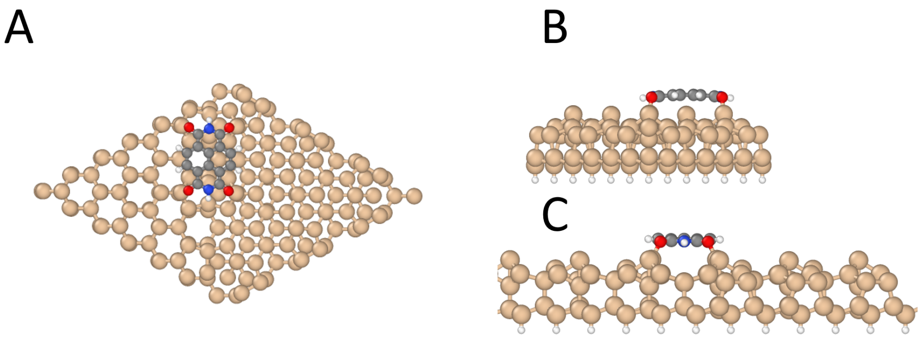

Supporting Figure 10: Potential adsorption geometry of NTCDI molecule on Si(111)-(7×7) surface in A) the (x, y) plane, B) (y, z) plane (cross-section of the long axis of the molecule), and C) (x, z) plane (cross-section of the short axis of the molecule).

The DFT calculated geometry that matched the most common configuration observed in experiment was taken from Jarvis et al. (2015) [19]. These images (see Fig.S10) were rendered in Ovito software [20].

From the atomic coordinates, we determined that the bowing in the long axis direction brings the nitrogen 20.2 *pm* below the central carbon-carbon double bond (C=C). The profile across the short axis was found to be inclined toward the right side by 3 *pm*.

Comparing Experimental and Simulated AFM

Experimental Data

The variation in height across the molecule was determined by calculating the average value in $z^*_{\Delta f}(x, y)$ (for the experimental data) and $z^*_F(x, y)$ (for the simulated data) along the points of interest across each of the axes of the molecule, i.e. either over a nitrogen atom, or a carbon-carbon double bond. The variance in height over each site was used to calculate the uncertainty.

From the experimentally measured C=C bond heights in Fig.4 (main text) we determined the adsorption geometry across the short axis to be flat, within error, for both 2D and 3D grids. The nitrogen atoms along the long axis dipped 11 ± 7 *pm* below the central carbon atoms according to the 3D grid, and 18 ± 9 *pm* below for the 2D grid.

Simulated Data

We applied the same methodology for finding height differences across the molecule to the simulated PPM data set. The total variation in height across the short axis was 3 ± 2 *pm*. The nitrogens along the long axis dip 18 ± 4 *pm* below the carbon atoms. This level of bowing calculated is in particularly good agreement with that of the 2D grid data set.

Tip Asymmetry

When performing $z^*(x, y)$ and $\Delta f^*(x, y)$ mapping, it is important to consider the effect of tip asymmetry. Similar to all SPM, these maps are necessarily a convolution of the structure of the sample (molecule) and the tip apex. Under the assumption of a uniform symmetric tip the $z^*(x, y)$ map provides an accurate approximation of the true molecular geometry (as demonstrated by the PPM simulation in Fig.4 from the main text for example). However, if the tip apex possesses a slight asymmetry this may affect the $z^*(x, y)$ map, and hence provide a distorted representation of the geometry.

We find our tips sometimes show a slight asymmetry in the attractive halo of the molecule during imaging, which suggests a tip asymmetry, however it is difficult to estimate the effect on the 3D data set from the halo present in a single CH image. Moreover, because we only restructure our tips via gentle indentation (rather than large scale indentation as is common in STM measurements), we find that for a given cantilever the slight asymmetry can often persist between measurements with nominally different tip terminations.

Typically, we find the $\Delta f^*(x, y)$ map (i.e. the 'depth' of the minima), is more strongly perturbed than the $z^*(x, y)$ map (i.e. the vertical position of the minima), which intuitively corresponds to the fact that the vertical position of the turning point is strongly governed by the onset of Pauli repulsion, which has an extremely strong distance dependence, compared to the more diffuse VdW forces which govern the depth of the minima. This is clearly

demonstrated in Fig.2 (main text), where, as discussed in the main text, the red curve demonstrates an abnormally small force minima, but the vertical position of the red curve minima is almost unperturbed relative to similar sites on the molecule.

Nonetheless, for tips with strong asymmetries we do observe distortion of the $z^*_{\Delta f}$ maps (as shown in Fig.S8). For this tip apex, it appears the slope on the tip apex in the short molecular axis direction is greater than the slope on the molecule (which is nearly flat), and so the $z^*_{\Delta f}$ map effectively provides an image of the tip apex rather than any tilt on the molecule. Along the short axis of the $z^*_{\Delta f}$ map there is an apparent tilt in the molecule, the rightmost carbon bond is 67 ± 10 pm below the leftmost, subtending an angle of approximately 7°. Given the flatness of the substrate around the molecule, the tilt observed in the molecule is likely due to the asymmetrical shape of the tip used for this particular experiment. This tilt is observed in both the topography and magnitude of force minima across the short axis of the molecule.

When discussing the results of the molecular geometry determination in the main text we ensured the z^* maps were taken with tips that did not show distortion due to tip asymmetry, but we note that a residual asymmetry could sometimes be observed in the Δf^* maps. For this reason when discussing the quantitative variation in the $F(z)$ minima taken on different sites (see below), we ensured these were compared along the long axis of the molecule, as the map, in this direction, did not show any evidence of tip asymmetry in either Δf^* or z^* maps.

Detector Noise

As discussed in Iwata et al. (2015) [1], the Δf signal itself is reduced by the use of large oscillation amplitudes, but the noise density of their deflection sensor was superior to an optimized room temperature qPlus system. From a thermal measurement of our commercial instrument [21], we estimate a high deflection noise of 600 $fm/\sqrt{Hz}$, which is in fact inferior to both the optical interferometer setup and an optimised qPlus sensor at room temperature (reported as 15 $fm/\sqrt{Hz}$ and 60 $fm/\sqrt{Hz}$ respectively, in [1]). It is also inferior to a commercial low temperature qPlus setup measured in our own laboratory (approx. 200 $fm/\sqrt{Hz}$). It is noteworthy that the relatively high detector noise of our commercial room temperature instrument did not inhibit our ability to obtain high-resolution intramolecular contrast, or high-resolution force maps. As we note in the main paper, we estimate the most significant source of noise in our measurement was the z stability of our microscope (excluding limited measurement time due to thermal stability as a factor).

Estimating Force Resolution

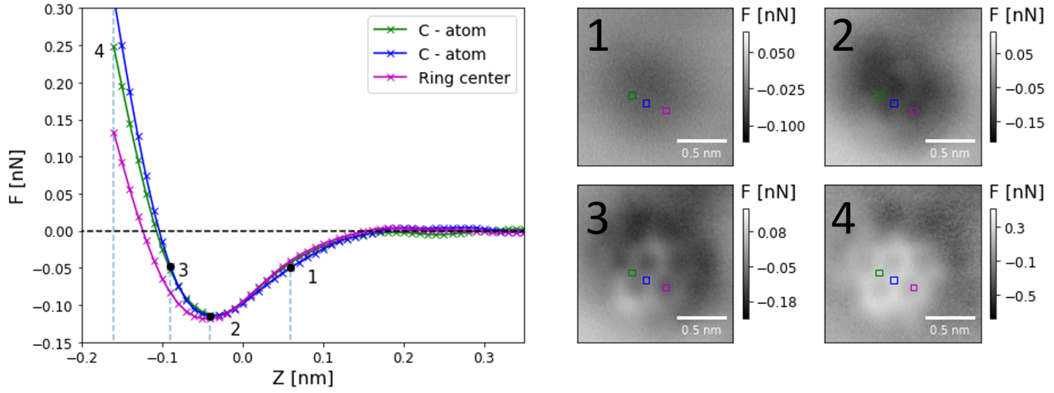

Supporting Figure 11: Extracted from the same data set as main text Fig.3. Left: force curves extracted over carbon atoms and center of nitrogen-containing ring of the molecule to estimate force resolution. Right: lateral positions of $F(z)$ curves indicated by square markers, which encompass the averaging window. The average difference in force minima between the ring and the points over the bond is $5pN$.

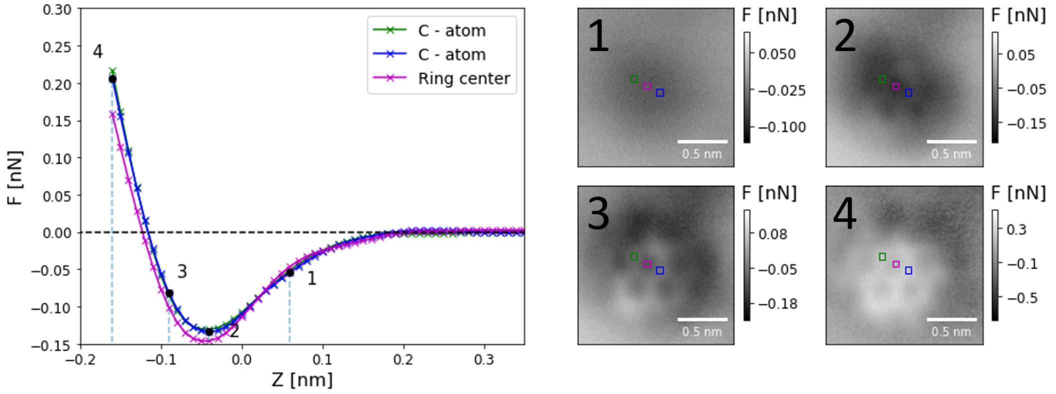

Supporting Figure 12: Extracted from the same data set as main text Fig.3. Left: force curves extracted over carbon atoms and benzene ring center site of the molecule to estimate force resolution. Right: lateral positions of $F(z)$ curves indicated by square markers, which encompass the averaging window. The average difference in force minima between the ring and the points over the bond is $14pN$.

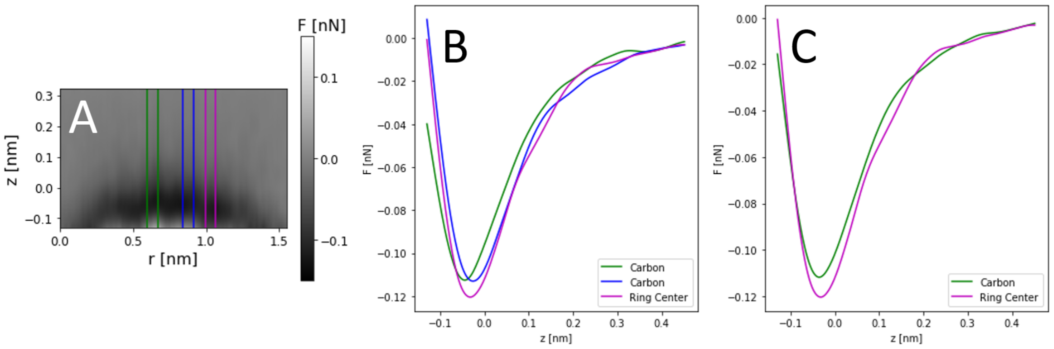

Supporting Figure 13: Estimating force resolution from main text Fig.2. A) Cross-sectional force map across long axis of molecule. B) Average single point $F(z)$ curves extracted over ranges indicated in A). C) Average of the blue and green force curves taken over bonds, for comparison to ring region force curves (purple). The difference in minima is $8pN$.

The force resolution exhibited by the data can be understood by determining the differences in minima across different sites of the molecule. Comparing the magnitude of force minima across the molecule must take into account the tip asymmetry that brings the signal more negative in Δf in the upper-right portion of the (x, y) plane. Hence, comparisons of magnitude (for the sake of estimating the force resolution) are made along lines that are as unaffected as possible by the shadow (i.e. parallel to the long axis). In Fig.S11, force minima over carbon atoms and the ring centers are extracted. The ring center has a force minimum of $-0.146\ nN$, and the carbon atoms have an average minimum $-0.132\ nN$.

This was repeated for another set of points parallel to the long axis in Fig.S12, an equivalent data set acquired with a different tip/molecule. There, the averaged minima are $-0.119\ nN$ and $-0.114\ nN$ for the ring and carbon atoms respectively. These same three points in (x, y) were examined using 2D grid data from Fig.2 (main text), and plotted in Fig.S13. The minima across the ring center (purple) and carbon atoms are $-0.120\ nN$ and $-0.112\ nN$ respectively. The average difference in the force minimum between atoms and ring centers across Fig.S11, S12, and S13 are $5\ pN, 14\ pN$, and $8\ pN$ respectively. From this we estimate our system is sensitive to variations in the tip-sample force on the order of $10\ pN$.

In Fig.2 (main text), at the equivalent central sites of the short and long 2D grids (at their intersection), we observed a difference in the force minima of $15\ pN$. Fig.S5 depicts an equivalent data set, acquired with a different tip and molecule. The force minima at the point of intersection between Fig.S5G and H, differ by $30\ pN$, and they are also displaced in z by $20\ pm$. Therefore while our measurements within a single grid are sensitive to force variations on the order of $\sim 10\ pN$, we can observe systematic uncertainties in the absolute force measured over equivalent sites between subsequent grids of up to $15 - 30\ pN$. There is good agreement between the force minima of equivalent sites between the 2D (Fig.S13) and 3D (Fig.3 in main text) grid experiments over the same molecule with the same tip. For example the force minima of the leftmost C=C bond along the short axis are $-69\ pN$ and $-68\ pN$ respectively, and for the rightmost nitrogen atoms along the long axis, $-146\ pN$ and $-130\ pN$, demonstrating a similar systematic uncertainty as with subsequent 2D grid measurements.

We note that DFT calculations from Iwata et al. (2015) [1] reported differences in force minima over PTCDA (a chemically similar molecule), between central carbon atoms and oxygen atoms of PTCDA molecules of 30 pN, assuming a silicon terminated tip. Across the carbon ring bonds and ring centres the difference in minima in their calculation was $\sim 50\ pN$. Our experimental data suggests a smaller difference, although this of course is dependent on the precise structure of the tip.

Dissipation

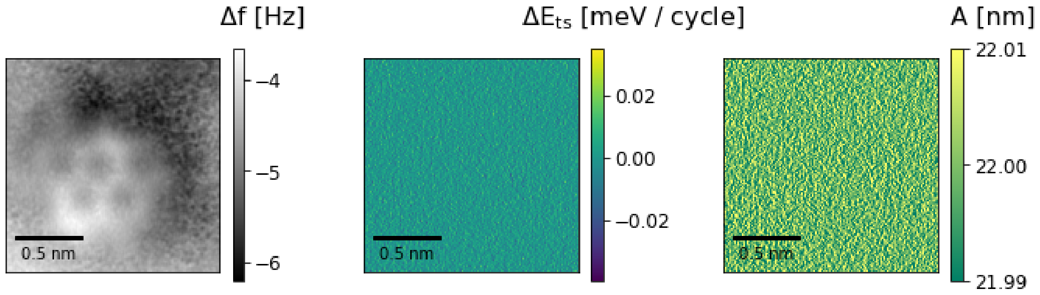

Supporting Figure 14: Raw Frequency shift, dissipation, and amplitude channels of a single scan from the lowest height scan in the 3D data set.

We include here in Fig.S14 an example of the dissipation and amplitude channels during the force mapping experiments. The dissipation image has been converted from the excitation channel (units of (V)) into the energy dissipation of the approach and retraction of the tip (units of $(meV\ /\ \text{cycle})$) using the equation for energy dissipation arising from the tip-sample interaction [13]:

$$\Delta E_{ts} = \frac{2\pi E_0}{Q}\left(\frac{A'_{drive}}{A_{drive}} - 1\right) \tag{3}$$

Where E_0 is the energy stored in the motion of the cantilever $(\frac{k}{2}A_0^2)$, A'_{drive} is the driving amplitude measured at the lowest slice, and A_{drive} is taken to be the $'$free$'$ damping signal, from the top slice. The dissipation and driving amplitude signals are negligible and so do not affect our force reconstruction. This is typical for our measurements of molecular systems at room temperature, and probably reflects the conservative nature of the interaction of a passivated tip with the molecule (similar to measurements with CO tips on molecules using a qPlus sensor at low temperature), which is different to the chemically reactive (e.g. silicon on silicon), or highly flexible (e.g. NaCl) systems often imaged using RT NC-AFM.

Positioning Error

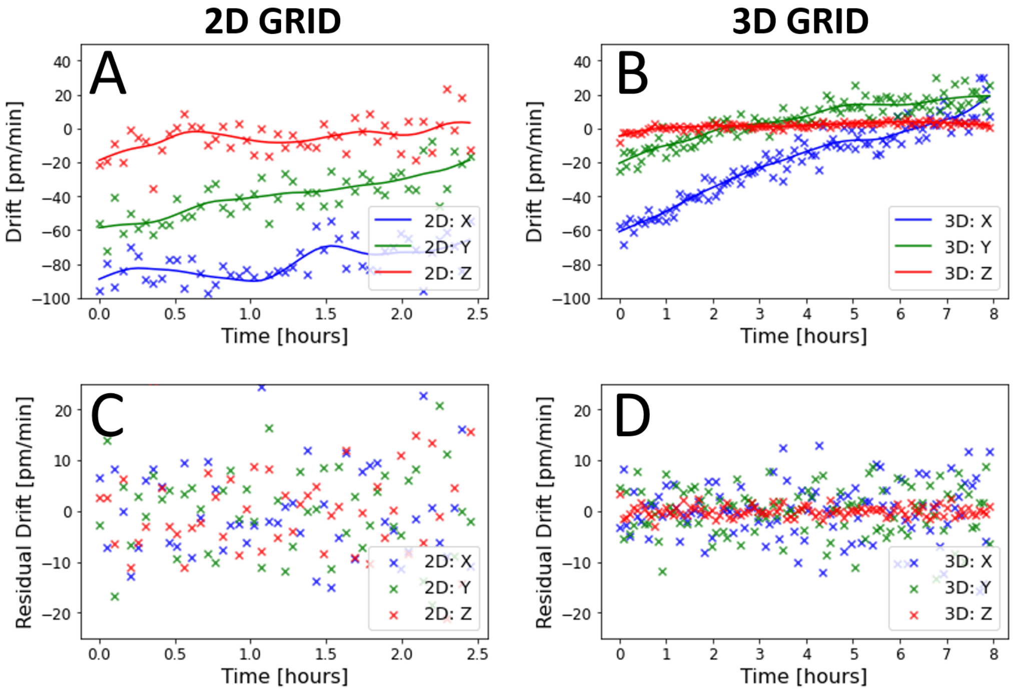

Supporting Figure 15: A) & B) Feedforward corrections made during 2D grid spectroscopy and 3D slice experiments respectively. C) & D) Corresponding residual drifts each time the feedforward vectors are updated for 2D and 3D experiments respectively.

The atom tracking, and hence, scripting, relies on the tip being able to reliably return to the same surface feature at each interval in the experimental measurement. This requirement poses an upper limit to the acquisition time t between the times where the tip is in feedback and tracking mode:

$$r_{drift}(t) - r_{drift}(0) < \Delta g \tag{4}$$

Where Δg is the desired precision of the lateral positioning [4]. The data in Fig.S15, depicts the feedforward corrective vectors applied to the scan-tube, hence an approximation the thermal drift between the tip and sample. The variation in drift is reduced by virtue of regulating the system temperature. Without regulation, the drift can vary on the order of 100 pm/min between each update of feedforward vectors, whereas the data across both 2D and 3D experiments in Fig.S15, (which were taken whilst regulating the temperature) exhibit variations on the order of 10 pm/min between each tracking event.

First, the residual drift of the 2D grid spectroscopy experiment C) had a maximum residual drift of 25 pm/min. This experiment updated the feedforward correction tracking after every 10 spectra, which totalled 180 seconds, hence the thermal drift may deviate from the compensated value, and displace from the intended position. Therefore, by the time the tip resumes tracking, a total maximum displacement of 75 pm along the x and y axes, may have occured. This equates to a maximum, radial displacement error of about 100 pm.

Second, D) shows the residual drift throughout 3D data set. The (x, y) plane showed a maximum residual drift of $\sim$ 15 pm/min, however in z, the maximum residual drift was smaller: $\sim$ 3 pm/min. Considering the displacement in the (x, y) plane, the acquisition time between instances of tracking = 270 seconds (which encompasses two image scans, as the script requires at least two measurements of the tracking position in order to make the displacement calculation), hence our maximum potential displacement along either axis is 67.5 pm, which gives a maximum displacement error of approximately 100 pm. Turning to the z-drift, this is corrected between each scan when it resumes feedback operation. Thus the effective acquisition time is 135 seconds. Therefore the maximum potential drift in z during a single scan is $\pm$7.5 pm, which is smaller than the 10 pm z resolution used in the experiment.

Alternatively, an estimate of the typical (rather of the maximum) error can be made using the median residual drift. For the 2D experiment, a typical error of 30 pm was observed in (x, y), and $\pm$8 pm in z. For the 3D experiment, a typical displacement error was 20 pm in (x, y), and $\pm$1.5 pm in z. Both data sets have similar pixel lateral pixel densities: 1 pixel $\sim$ 5 pm. The z-resolutions of the 2D and 3D grids were 1.37 pm and 10 pm respectively.

The drift correction of the slice method exhibited better performance over the 2D grid spectroscopy method, despite having a larger acquisition time. As these experiments were carried out consecutively, it is unlikely the external environment was the reason for the difference in thermal drift values. A possible explanation for the poorer performance of the grid spectroscopy could be the piezo-creep. After moving distances of the order of 1 nm in z, the tip resumes feedback and tracking, and the piezo crystal may be still relaxing during the measurement of its average position. We note that Rahe et al. (2011) [4] reported positioning precision, Δg of approximately 50 pm, which exceeds our estimates above.

References

(1) Iwata, K.; Yamazaki, S.; Mutombo, P.; Hapala, P.; Jelínek, P.; Sugimoto, Y. *Nature Communications* **2015**, *6*, 7766.

(2) Abe, M.; Sugimoto, Y.; Custance, Ó.; Morita, S. *Nanotechnology* **2005**, *16*, 3029–3034.

(3) Fremy, S.; Kawai, S.; Pawlak, R.; Glatzel, T.; Baratoff, A.; Meyer, E. *Nanotechnology* **2012**, *23*.

(4) Rahe, P.; Schtte, J.; Schniederberend, W.; Reichling, M.; Abe, M.; Sugimoto, Y.; Khnle, A. *Review of Scientific Instruments* **2011**, *82*.

(5) Sugimoto, Y.; Nakajima, Y.; Sawada, D.; Morita, K.-i.; Abe, M.; Morita, S. *Physical Review B* **2010**, *81*, 245322.

(6) Sweetman, A. M.; Jarvis, S. P.; Sang, H.; Lekkas, I.; Rahe, P.; Wang, Y.; Wang, J.; Champness, N. R.; Kantorovich, L.; Moriarty, P. *Nature Communications* **2014**, *5*, 3931.

(7) Nečas, D.; Klapetek, P. *Central European Journal of Physics* **2012**, *10*, 181–188.

(8) Moreno, C.; Stetsovych, O.; Shimizu, T. K.; Custance, Ó. *Nano Letters* **2015**, *15*, 2257–2262.

(9) Sweetman, A.; Rashid, M.; Jarvis, S.; Dunn, J. L.; Rahe, P.; Moriarty, P. *Nature Communications* **2016**, *7*, 10621.

(10) Monig, H.; Amirjalayer, S.; Timmer, A.; Hu, Z.; Liu, L.; Arado, O. D.; Cnudde, M.; Strassert, C. A.; Ji, W.; Rohlfing, M.; Fuchs, H. *Nature Nanotech* **2018**, 371–375.

(11) Gross, L.; Mohn, F.; Moll, N.; Liljeroth, P.; Meyer, G. *Science* **2009**, *325*, 1110–1114.

(12) Giessibl, F. *Review of Scientific Instruments* **2019**, *90*, 011101.

(13) Giessibl, F. J. *Reviews of Modern Physics* **2003**, *75*, 949–983.

(14) Ternes, M.; González, C.; Lutz, C. P.; Hapala, P.; Giessibl, F. J.; Jel inek; Heinrich, A. J. *Phys. Rev. Lett* **2011**, *106*, 16802.

(15) Sader, J.; Jarvis S, P. *Appl. Phys. Lett.* **2004**, *84*, 1801–1803.

(16) Gross, L.; Schuler, B.; Liu, W.; Tkatchenko, A.; Moll, N.; Meyer, G.; Mistry, A.; Fox, D. *Physical Review Letter* **2013**, *111*, 106103.

(17) Mohn, F.; Gross, L.; Meyer, G. *Applied Physics Letters* **2011**, *99*, 53106.

(18) Hapala, P.; Kichin, G.; Wagner, C.; Tautz, F. S.; Temirov, R.; Jelínek, P. *Phys. Rev. B* **2014**, *90*, 085421.

(19) Jarvis, S. P.; Sweetman, A. M.; Lekkas, I.; Champness, N. R.; Kantorovich, L.; Moriarty, P. *Journal of Physics Condensed Matter* **2015**, *27*, 054004.

(20) Stukowski, A. *Modelling and Simulation in Materials Science and Engineering* **2009**, *18*, 015012.

(21) Lubbe, J.; Temmen, M.; Rode, S.; Rahe, P.; Kuhnle, A.; Reichling, M. *Nanotechnol.* **2013**, *4*, 32–44.
